# Epigallocatechin Gallate Protects against MNNG-Induced Precancerous Lesions of Gastric Carcinoma in Rats via PI3K/Akt/mTOR Pathway

**DOI:** 10.1155/2021/8846813

**Published:** 2021-02-05

**Authors:** Feiye Zhu, Yanli Xu, Jieli Pan, Meiya Li, Fangming Chen, Guanqun Xie

**Affiliations:** ^1^Academy of Chinese Medical Sciences, Zhejiang Chinese Medical University, Hangzhou, Zhejiang 310053, China; ^2^The First Affiliated Hospital of Zhejiang University School of Medicine, Hangzhou, Zhejiang 310003, China; ^3^College of Basic Medical Science, Zhejiang Chinese Medical University, Hangzhou, Zhejiang 310053, China

## Abstract

**Objective:**

To evaluate the therapeutic effect of epigallocatechin gallate (EGCG) on precancerous lesions of gastric carcinoma (PLGC) and to determine whether EGCG protects against PLGC by regulating PI3K/Akt/mTOR pathway.

**Methods:**

Twenty-four male Wistar rats were randomly divided into 3 groups: normal control group (NC), PLGC model group (MC), and group of PLGC rats treated with EGCG (MC + EGCG). 1-Methyl-3-nitro-1-nitrosoguanidine (MNNG) and sodium salicylate were combined and used to establish the PLGC rat animal model. The therapeutic effect of EGCG on PLGC was evaluated by body weight and pathological lesions of gastric mucosa in PLGC rats. Quantitative polymerase chain reaction (qPCR) was applied to measure the mRNA expressions of PI3K, Akt, and mTOR. The protein expressions of cleaved caspase-3, PTEN, PI3K, p-PI3K, Akt, p-Akt, p-mTOR, and mTOR were determined by automated western immunoblotting.

**Results:**

The body weight decreased in PLGC rats while EGCG significantly increased body weight. The gastric mucosa of PLGC rats exhibited the pathological lesions of atrophy, intestinal metaplasia, and atypical hyperplasia while EGCG could ameliorate the pathological lesions. EGCG could upregulate the expressions of cleaved caspase-3 and PTEN and reduce the expressions of PI3K, Akt, and mTOR.

**Conclusions:**

EGCG ameliorated pathological lesions of PLGC and exerted the effect of apoptosis promotion in PLGC rats. The apoptotic pathway triggered by EGCG may be related to inhibition of PI3K/Akt/mTOR pathway. It provided a theoretical basis for the PLGC treatment and gastric cancer prevention.

## 1. Introduction

Gastric cancer, the fifth most common cancer, is responsible for the third leading cause of cancer deaths worldwide [1]. Despite multitherapy, more than 60% of patients experience recurrence or metastasis. The overall 5-year survival rate of patients with gastric cancer is less than 30% in the world; especially those with advanced gastric cancer has a worse prognosis of less than 20% [2]. Therefore, early detection and treatment are crucial for the prevention of gastric cancer. The progression of the normal gastric mucosal cells into gastric cancer is multistep, including normal epithelia, nonatrophic gastritis, atrophic gastritis, intestinal metaplasia, dysplasia, and cancer [3]. Precancerous lesions of gastric carcinoma (PLGC) are a gastric mucosal pathology state of atrophic gastritis with intestinal metaplasia and dysplasia. PLGC serves a prominent role in preventing gastric cancer in its early stage [4].

Green tea is a popular healthy drink in the world. Its health benefits are associated with reduced risk of many chronic diseases, such as different cancers, diabetes, and cardiovascular diseases [5]. Epigallocatechin gallate (EGCG) is a major polyphenolic constituent derived from green tea and has significant anticarcinogenic, antioxidant, anti-inflammatory, and neuroprotective properties [6]. Extensive research on EGCG has brought into light their potential to prevent cancer by inducing apoptosis and suppressing cell proliferation, migration, and invasion [7, 8]. In addition, it has been reported that EGCG can protect vascular endothelial cells from oxidative stress-induced damage [9]. Studies on the anticancer activity of EGCG focus on various types of cancer, including lung, breast, prostate, ovarian, colorectal, and esophageal; however, few studies focus on whether EGCG can induce apoptosis of precancerous cells in lesions of gastric mucosa in PLGC [10–15].

PI3K/Akt/mTOR pathway is involved in cancer tumorigenesis, proliferation, and progression which makes this pathway crucial in cancer therapy [16, 17]. Previous studies indicated that EGCG can treat a variety of cancers by inhibiting this signaling pathway [18, 19], but it was not verified if EGCG exerted mechanism in the treatment of PLGC by targeting PI3K/Akt/mTOR pathway. Thus, we hypothesized that EGCG would treat PLGC by regulating PI3K/Akt/mTOR pathway. In the present study, we established the PLGC rat model to investigate the molecular mechanism of EGCG and to support the clinical application of EGCG in the treatment of PLGC.

## 2. Materials and Methods

### 2.1. Animals

Twenty-four male Wistar rats (body weight 120∼140 g) were purchased from Shanghai Slac Laboratory Animal Co. Ltd. (Shanghai, China) and bred at Laboratory Animal Research Center of Zhejiang Chinese Medical University (Hangzhou, China). All the rats were housed in an SPF controlled environment (room temperature 23 ± 1°C; humidity 55∼60%) with a 12 h light/dark cycle and free access to food and water. The animal experimental protocol was approved by the Animal Welfare Committee of Zhejiang Chinese Medical University, Hangzhou, China (SYXK2018-0012).

### 2.2. Drugs and Reagents

EGCG (purity > 99%) was purchased from Chengdu Must Bio-Technology Co., Ltd. (Chengdu, China). 1-Methyl-3-nitro-1-nitrosoguanidine (MNNG) was provided by Tokyo Chemical Industry Co., Ltd. (Tokyo, Japan). Sodium salicylate was purchased from Shanghai Xilong Biochemical Technology Co., Ltd. (Shanghai, China). Total RNA Rapid Extraction Kit, HiFiScript Quick gDNA Removal cDNA kit, and SYBR green PCR Master Mix were obtained from Beijing Biotek Biotechnology Co., Ltd. (Beijing, China). The primers used for quantitative polymerase chain reaction (qPCR) were synthesized by the Sangon Biotech (Shanghai) Co., Ltd. (Shanghai, China). RIPA buffer and BCA protein quantification kit were ordered at Beyotime Biotechnology (Shanghai, China). The primary antibodies included anticleaved caspase-3 (9664, CST, USA), anti-PTEN (9559, CST, USA), anti-Akt (4691, CST, USA), anti-p-Akt (4060, CST, USA), anti-mTOR (2983, CST, USA), anti-p-mTOR (5536, CST, USA), anti-GAPDH (5174, CST, USA), anti-PI3K (ab227204, Abcam, UK), and anti-p-PI3K (ab182651, Abcam, UK). The Size Separation Master Kit with Split Buffer (12–230 kDa/66–440 kDa) was obtained from ProteinSimple (California, USA)

### 2.3. Animal Experiment

A total of twenty-four rats were randomly divided into 3 groups (*n* = 8 per group): normal control group (NC), PLGC model group (MC), and group of PLGC rats treated with EGCG (MC + EGCG). Except for the NC rats, others were given 100 *μ*g/mL of MNNG solution protected from light for free drink and orally administrated 2 ml of 2% (M/V) sodium salicylate twice a week. The NC rats had free access to water. The procedure lasted for 17 weeks. After that, rats in the MC + EGCG group were orally administered with EGCG at a dosage of 50 mg/kg once daily from the 18^th^ to the 21^st^ week, and at the same time, the NC and MC rats were administered 0.9% saline. The body weight of each was measured once a week. At the end of the experiment, rats were sacrificed by intraperitoneal injection of sodium pentobarbital (50 mg/kg) and blood samples were collected and centrifuged at 3000 rpm for 15 min to obtain serum. Gastric tissues were cut open on ice along the greater curvature and the gastric antrum was divided into three parts. One was fixed in 10% formalin solution for histological study and AB-PAS staining. Another was kept in 2.5% glutaraldehyde for ultrastructure examination and the final was stored at −80°C for automated western immunoblotting and qPCR analysis.

### 2.4. Histological Evaluation

The morphological changes were analyzed with hematoxylin and eosin (H&E) staining. Gastric tissues of the rats were fixed with 10% formalin and then dehydrated with a serious of concentrations of ethanol and xylene. Dehydrated samples were embedded in paraffin, cut into sections (5 *μ*m), and then stained with H&E. The different types of intestinal metaplasia were detected by AB-PAS staining which was performed according to the manufacturer's instruction. The images of stained sections were acquired using an automatic photomicroscope (Nikon, Japan).

### 2.5. Ultrastructural Examination

The 1 cm × 1 cm × 5 mm gastric tissues of rats were fixed with 2.5% glutaraldehyde at 4°C overnight and then stained with 1% osmic acid at room temperature for 1.5 h. Dehydration of tissues was carried out using a series of concentrations of ethanol (50%, 75%, 90%, and 100%) and tert-butanol (50%, 75%, 90%, and 100%). Each tissue was freeze-dried with a freeze dryer. The ion sputtering instrument (Hitachi, Japan) was used to spray gold for 30 s. The ultrastructure of gastric tissues was observed using a Hitachi SU8010 scanning electron microscope (SEM) (Hitachi, Japan).

### 2.6. RNA Extraction and qPCR

Total RNA was extracted from gastric tissues using Total RNA Rapid Extraction Kit. RNA purity was measured with a Nanodrop one (Thermo Scientific, USA). cDNA was synthesized with a HiFiScript Quick gDNA Removal cDNA kit. SYBR green PCR Master Mix was used for qPCR (Roche, Roche 480 real-time system). Primer sequences are shown in Table 1. *β*-Actin was used as a reference standard. The reaction conditions were 95°C 10 min; 95°C 10 s; 60°C 30 s, 72°C 32 s, 45 cycles; melting curve analysis was at 65°C∼97°C. Relative mRNA expression was calculated and analyzed by the 2^−△△Ct^ method.

### 2.7. Protein Extraction and Automated Western Immunoblotting

The gastric tissues were homogenized with RIPA buffer containing protease and phosphatase inhibitor. Then, the protein content was quantified using a BCA protein quantification kit. A Peggy Sue system (ProteinSimple, California, USA) was used to perform simple western immunoblotting according to the manufacturer's instruction of the Size Separation Master Kit with Split Buffer (12–230 kDa/66–440 kDa). Compass software (version 2.7.1, Protein Simple) was used to program the Peggy Sue and for the presentation of the western immunoblotting. GAPDH was used as an internal control.

### 2.8. Determination of Hepatotoxicity and Nephrotoxicity

Serum alanine aminotransferase (ALT), aspartate aminotransferase (AST), and total bilirubin (TBIL) were used as the biochemical markers of hepatotoxicity and serum creatinine (CREA) and blood urea nitrogen (BUN) were used for evaluating the nephrotoxicity of EGCG. All of the biochemical markers were detected using an autobiochemical analyzer (Hitachi, Japan).

### 2.9. Statistical Analysis

Measurement data were presented as mean ± SEM and analyzed by SPSS 16.0 software. The statistical differences among experimental groups were evaluated by the Student's *t*-test or Mann–Whitney *U* test when appropriate. A value of *P* < 0.05 was accepted as statistically significant. GraphPad Prism 5.0 software was used to make the corresponding figures.

## 3. Results

### 3.1. EGCG Improved the Growth of PLGC Rat

**Figure 1:**
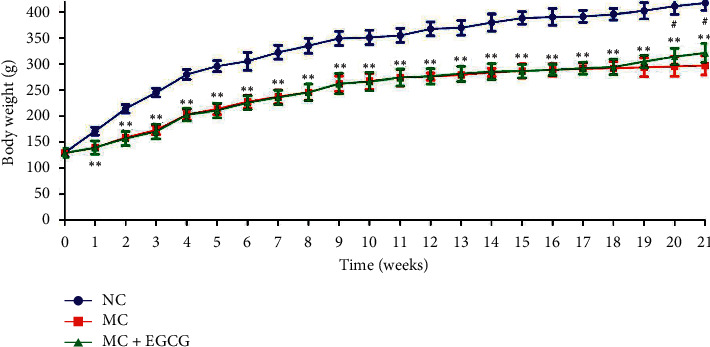
The effect of EGCG on the body weight of the PLGC rat. The body weight was decreased in the PLGC rats, while it increased after EGCG treatment. Data are expressed as mean ± SEM. ^*∗∗*^*P* < 0.01 versus NC group. ^*#*^*P* < 0.05 versus MC group.

To evaluate the effect of EGCG on the growth of PLGC rats, the body weight was recorded every week. As shown in Figure 1, the body weight of the NC rats regularly increased with each passing week. The body weight of the MC rats, however, rose up at a noticeably slower pace, and just after the first week, the body weight of the MC group was significantly lower than that of the NC group (*P* < 0.01). The rats treated with EGCG gained more weight than those in the MC group by the 20^th^ week (*P* < 0.05).

### 3.2. EGCG Alleviated the Degree of Gastric Mucosal Lesions

**Figure 2:**
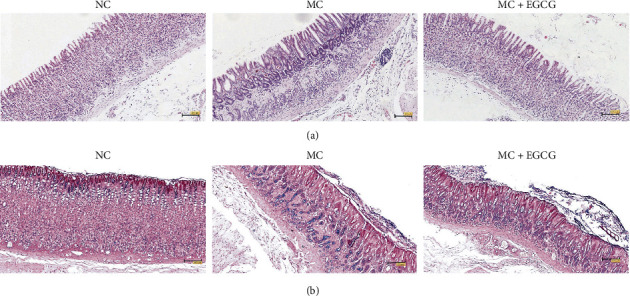
EGCG alleviated the degree of gastric mucosal lesions. (a) The rat gastric tissue was collected, and the pathological changes were observed after the H&E staining. Bar = 100 *μ*m. (b) The establishment of the PLGC model and the effect of EGCG on the gastric mucosa of PLGC rats were further evaluated by AB-PAS staining. Bar = 100 *μ*m.

In order to investigate the protective effect of EGCG on the gastric mucosa of PLGC rats, the H&E staining was performed. The gastric mucosa of NC rats was intact, the glands were arranged regularly, the muscular layer of mucosa was thin, and a small number of lymphocytes were scattered in the lamina propria. Compared with the NC group, the gastric mucosa of PLGC rats was obviously atrophic and thin, the glands were significantly reduced, and the arrangement was irregular. The muscular layer of mucosa was thicker and extended to the lamina propria, which was inserted into the glands in a branched manner, and a large number of lymphocytes infiltrated in the stroma. Some rats showed intestinal metaplasia, atypical hyperplasia, and some nuclear fission, which indicated that the PLGC model has been successfully established. After administration of EGCG, the mucosa was basically intact, the glands were arranged regularly, the muscular layer of mucosa was thin, and a few lymphocytes infiltrated in the stroma. The degree of intestinal metaplasia was reduced, and atypical hyperplasia was rare or not found (Figure 2(a)).

AB-PAS staining is a generally recognized method to judge the PLGC model, which is used to further evaluate the establishment of the PLGC model. The upper of gastric mucosa in the NC group was positive and dyed red by PAS staining, while the blue layer in the middle and bottom sections by AB staining was not obvious. In the MC group, the middle and bottom sections were positive and dyed blue by AB staining, indicating an acidic mucus secreted by goblet cells. PAS was weakly positive in the upper part, with only a small amount of red staining. After treatment with EGCG, AB positive staining at the middle and bottom decreased, and the blue layer was also thinned. PAS staining in the upper was positive, and the red layer became thicker (Figure 2(b)).

### 3.3. EGCG Repaired Gastric Mucosal Ultrastructural Lesions

**Figure 3:**
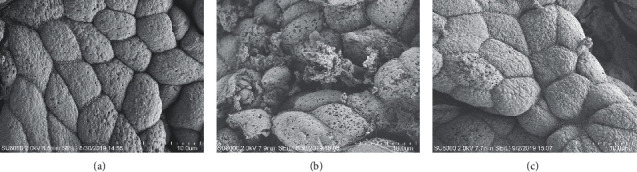
EGCG repaired gastric mucosal ultrastructural lesions. The degree of gastric mucosal ultrastructural lesions was judged by SEM. The ultrastructure of gastric mucosa in PLGC rats was damaged, while EGCG could repair the gastric mucosal ultrastructural lesions. Magnification was 3.5 K. Bar = 10 *μ*m. (a) NC. (b) MC. (c) MC + EGCG.

The degree of gastric mucosal ultrastructural lesions was judged by SEM. The epithelial cells of gastric mucosa were closely connected and arranged regularly with the complete structure in the NC group. Compared with the NC group, the epithelial cells of PLGC rats were atrophy and irregular arrangement. The epithelial cells ruptured and shed, leading to focal mucosal injury. After treatment with EGCG, the atrophy of the epithelial cells was mild and with little rupture (Figure 3).

### 3.4. EGCG Induced Cell Apoptosis in PLGC Rats

**Figure 4:**
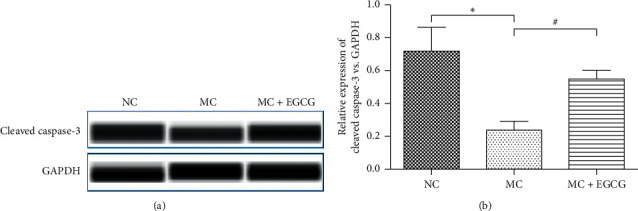
EGCG induced cell apoptosis in PLGC rats. The protein expression of cleaved caspase-3 (a) and (b) in the MC group decreased compared with that in the NC group, while it increased after EGCG treatment. Data are expressed as mean ± SEM. ^*∗*^*P* < 0.05 versus NC group. ^#^*P* < 0.05 versus MC group.

To explore the EGCG intervention mechanism in PLGC and to make clear whether it associated with apoptosis, apoptosis protein cleaved caspase-3 in gastric mucosa of rats was detected. As shown in Figure 4, the protein expression of cleaved caspase-3 in MC group was lower than that in the NC group (*P* < 0.05). EGCG treatment upregulated the protein expression of cleaved caspase-3 significantly (*P* < 0.05) which indicated that EGCG promoted cell apoptosis in PLGC rats.

### 3.5. EGCG Inhibited the Activation of PI3K/Akt/mTOR Pathway in PLGC Rats

**Figure 5:**
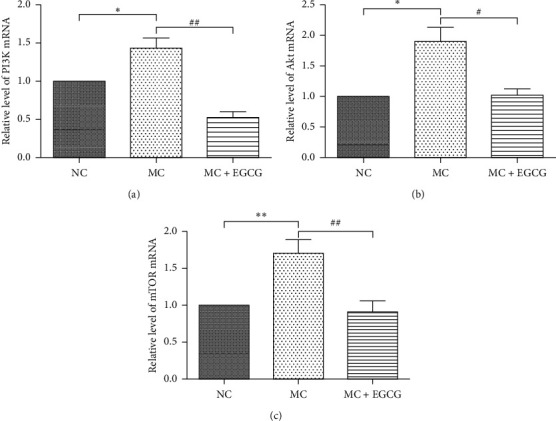
EGCG inhibited the mRNA levels of PI3K, Akt, and mTOR. The mRNA expressions of PI3K (a), Akt (b), and mTOR (c) in the MC group increased compared with that in the NC group, while they decreased after EGCG treatment. *β*-Actin was used as an internal control. Data are expressed as mean ± SEM. ^*∗*^*P* < 0.05, ^*∗∗*^*P* < 0.01 versus NC group. ^#^*P* < 0.05, ^##^*P* < 0.01 versus MC group.

**Figure 6:**
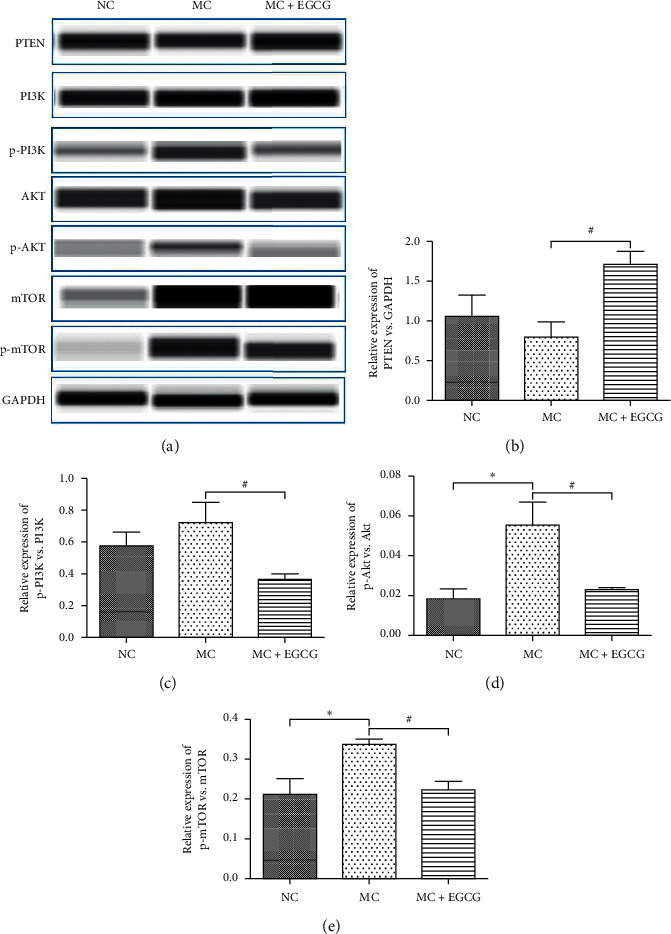
The effect of EGCG on the protein expressions of PTEN, PI3K, Akt, and mTOR. The protein expression of PTEN (a, b) in the MC group decreased compared with that in the NC group, while it increased after EGCG treatment. The protein expressions of PI3K (a, c), Akt (a, d), and mTOR (a, e) in the MC group increased compared with that in the NC group, while they decreased after EGCG treatment. Data are expressed as mean ± SEM. ^*∗*^*P* < 0.05 versus the NC group. ^#^*P* < 0.05 versus the MC group.

PI3K/Akt/mTOR pathway is involved in the progression of gastric cancer. To further determine the effect of EGCG on the PI3K/Akt/mTOR pathway in PLGC rats, we measured the mRNA and protein expressions of factors related to the pathway. The mRNA levels of PI3K, Akt, and mTOR in gastric mucosa of PLGC rats significantly increased (*P* < 0.05 or *P* < 0.01). Compared to the MC group, the expressions of PI3K, Akt, and mTOR markedly reduced after EGCG administration (*P* < 0.05 or *P* < 0.01) (Figure 5). The protein expressions of PI3K, Akt, and mTOR exhibited the same tendency as the mRNA levels (Figures 6(a), 6(c)–6(e)). As a negative regulator of the pathway, PTEN could be upregulated by EGCG (*P* < 0.05) (Figures 6(a) and 6(b)). These findings demonstrate that EGCG inhibits the activation of the PI3K/Akt/mTOR pathway in PLGC rats.

### 3.6. Effect of EGCG on Liver and Kidney Function in PLGC Rats

**Figure 7:**
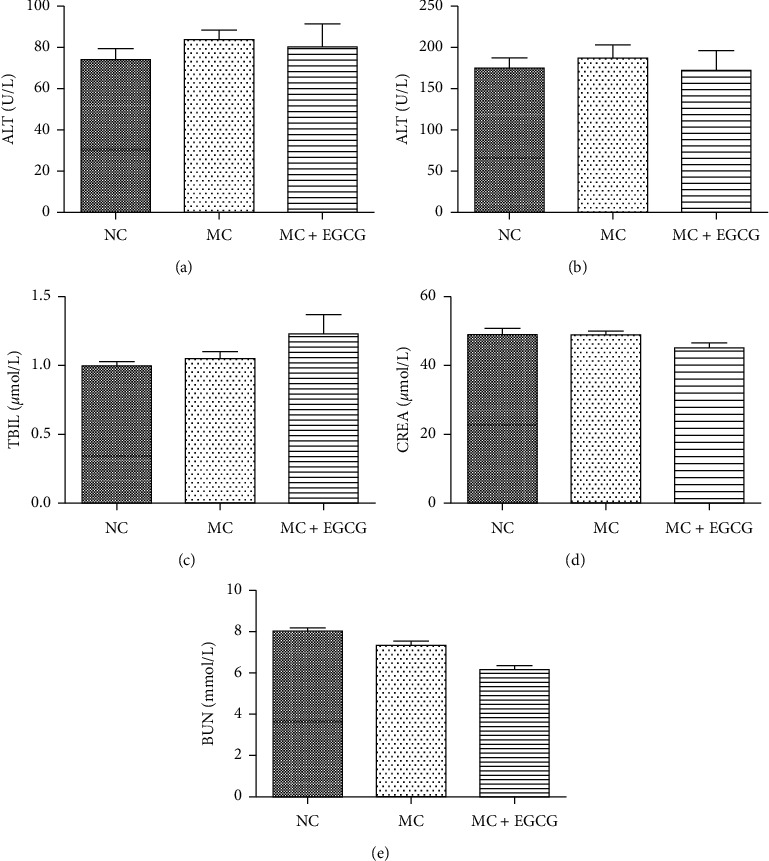
The effect of EGCG on serum ALT, AST, TBIL, CREA, and BUN. The levels of serum ALT (a), AST (b), TBIL (c), CREA (d), and BUN (e) were evaluated by the autobiochemical analyzer. Data are expressed as mean ± SEM.

To evaluate the safety and toxicity of EGCG, we measured levels of indicators in peripheral blood, including ALT, AST, TBIL, CREA, and BUN. As shown in Figure 7, treatment with EGCG did not significantly alter the levels of serum ALT, AST, TBIL, CREA, and BUN.

## 4. Discussion

PLGC is the early stage of gastric cancer. The annual incidence of gastric cancer was 0.6% for mild to moderate dysplasia and 6% for severe dysplasia [20]. Therefore, diagnosis and treatment in the early stage are important strategies to prevent gastric cancer. Since precancerous lesions are reversible while there are no efficacious drugs for it at present, it is of great significance to explore effective drugs for the treatment of PLGC [21].

EGCG is a key polyphenolic component in green tea and exerts a significant effect on anticancer [22], but its influence on PLGC is still unknown. Thus, it is of great significance and value to study the effect of EGCG on PLGC and explore the underlying molecular mechanism. As a natural product and dietary supplement, EGCG is considered safe and can be taken for a long time. However, a study reported that the high concentration of EGCG could lead to hepatotoxicity [23]. The low concentration of EGCG could rescue liver failure caused by hepatotoxicity [24]. Therefore, it is crucial to select a safe and effective dose for this study. A study reported that the observed safe level of 704 mg/EGCG might be considered for tea preparations in beverage [25]. Thus, we chose a dosage below a safe level and determined the dosage of rats in this experiment was 50 mg/kg/d according to the equivalent dose conversion of the human body and rat. In the current study, we first aimed to investigate the effect of the low dose of EGCG on PLGC.

Gastric cancer is a common malignant gastrointestinal tumor. Its occurrence is due to multiple factors, and its development presents a multistep progressive pathological process [26]. Unhealthy diet, especially preserved food containing high amounts of N-nitroso compounds, plays a curial role in the occurrence and development of gastric cancer [27]. As an activated N-nitroso compound, MNNG simulates the process of carcinogens in the stomach caused by improper intake of nitrate and is currently used in the establishment of an animal model of PLGC [28]. Sodium salicylate, a nonsteroidal anti-inflammatory drug, is another substance for inducing the PLGC model due to the fact that it causes an inflammatory reaction of gastric mucosa through direct stimulation of local gastric mucosa, resulting in the shedding of gastric mucosal cells [29]. Therefore, the combination of MNNG and sodium salicylate is widely used to simulate the carcinogenesis process of the normal gastric mucosa. In this study, MNNG and sodium salicylate were combined and used to establish the PLGC rat animal model. The gastric mucosa of PLGC rats exhibited the pathological lesions of atrophy, intestinal metaplasia, and atypical hyperplasia, indicating the PLGC animal model was successfully established. After treated with EGCG, the body weight increased and pathological lesions of PLGC rats were ameliorated significantly, suggesting that treatment of EGCG at the low dose was effective. In addition, we found that there was no obvious hepatotoxicity and nephrotoxicity in PLGC rats treated with EGCG by detecting the serum levels of ALT, AST, TBIL, CREA, and BUN in this study. Therefore, we speculate that low-dose EGCG is safe and effective for PLGC, which is worthy of further research.

Cell apoptosis plays a negative regulatory role in the development of the tumor, which can inhibit the rapid growth of tumor cells [30]. Apoptosis-related protease caspase is activated with cell apoptosis. Caspase-3 is one of the caspase family that directly induces apoptosis [31]. Recent studies have shown that EGCG can promote tumor cell apoptosis through various signaling pathways [32]. In this study, EGCG upregulated the expression of cleaved caspase-3 and was confirmed to promote apoptosis in PLGC rats.

PI3K/Akt/mTOR pathway is a classic signal pathway of apoptotic regulation, which can regulate multiple apoptosis-related proteins or families [33]. Abnormal activation of this pathway can promote the abnormal proliferation and differentiation of tumor cells, inhibit cell apoptosis, enhance cell tolerance to hypoxia and malnutrition, and promote tumor cell metastasis [34, 35]. The tumor suppressor PTEN is a negative regulator of the PI3K/Akt/mTOR signaling by converting PIP3 to PIP2 and preventing phosphorylation of Akt, further inhibiting the phosphorylation of mTOR [36]. Thus, upregulation of PTEN can promote cell apoptosis. The present study demonstrated that the expression of PTEN increased after EGCG treatment in PLGC rats, while the expressions of PI3K, AKT, and mTOR reduced, suggesting EGCG may reverse PLGC through the PI3K/Akt/mTOR pathway.

The occurrence of gastric cancer is a typical representative of the transformation from inflammation to cancer. PLGC, an important stage of inflammation and cancer transformation, is a refractory precancerous state. At present, there is a lack of safe and effective treatment drugs to block this process. EGCG is extracted from the health drink of green tea. Previous studies have confirmed that EGCG has anticancer, anti-inflammatory, and antioxidation effects. Our study shows that low-dose EGCG has a good curative effect and high safety for PLGC. Therefore, EGCG has important clinical significance and good prospects in the treatment of PLGC. However, EGCG is a multitarget drug, and the mechanism of its treatment of PLGC has not been fully elucidated. We will further study EGCG through cell experiments, gene knockout, modern nanopackaging technology, and drug molecular technology to make EGCG have better clinical application value.

## 5. Conclusions

In conclusion, EGCG ameliorated pathological lesions of PLGC and exerted the effect of apoptosis promotion in PLGC rats. The apoptotic pathway triggered by EGCG may be related to the inhibition of the PI3K/Akt/mTOR pathway, which was one of the mechanisms of EGCG for the treatment of PLGC. The present study provided a theoretical basis for the treatment of PLGC. Moreover, our study helps to obtain an important clue for the potential application of EGCG on PLGC therapy and gastric cancer prevention.

## Figures and Tables

**Table 1:** Primer sequences for RT-qPCR.

Gene	Description	Sequence (5′-3′)
Rat PI3K	Forward	CCGAAAGTTCAGGGTCA
	Reverse	AGGAAGCGGTGGTCTAT

Rat Akt	Forward	GAGGAGCGGGAAGAGTG
	Reverse	TGCCCTTGCCCAGTAG

Rat mTOR	Forward	AGATACGCCGTCATTCCT
	Reverse	GCTCAAACACCTCCACCT

Rat *β*-actin	Forward	CGTGCGTGACATTAAAGAG
	Reverse	CTGGAAGGTGGACAGTGAG

## Data Availability

All datasets analyzed to support the findings of the current study are available from the corresponding author upon reasonable request.
